# Phosphorus – a scoping review for Nordic Nutrition Recommendations 2023

**DOI:** 10.29219/fnr.v67.10318

**Published:** 2023-12-20

**Authors:** Suvi T. Itkonen, Christel Lamberg-Allardt

**Affiliations:** Department of Food and Nutrition, University of Helsinki, Helsinki, Finland

**Keywords:** phosphorus, phosphate, phytate, hydroxyapatite, nutrition recommendations

## Abstract

This scoping review aims to describe the totality of evidence for the role of phosphorus for health-related outcomes as a basis for setting and updating dietary reference values (DRVs). Phosphorus is needed in many biological processes, such as cellular metabolism and bone mineralization. Dietary phosphorus intake exceeds the previous Nordic Nutrition Recommendations (NNR2012) 2–3-fold in the Nordic countries. Intake from food additives is unknown but may play a significant role because the use of phosphate additives is common in the food industry. Bioavailability of phosphorus in plant-based products is lower than animal-based products. Nevertheless, bioavailability of phosphorus additives is higher. The main phosphorus-related health outcomes concern high phosphorus intake mainly from food additives with potential adverse effects on kidney, bone, and cardiovascular health.

## Popular scientific summary

- Phosphorus is an essential element needed for many biological molecules and processes, such as cellular metabolism and bone mineralization.
- The main food sources of phosphorus in the Nordic countries are dairy products, cereal products, and meat products.
- Deficiency is very rare but can cause skeletal defects and problems with the nervous system, muscle tissue, and kidney function.
- No reliable biomarker of phosphorus intake and status has been established.
- More data on bioavailability, phosphate content in foods, long-term health outcomes, and risk of chronic diseases are needed.

The aim of this scoping review is to describe the totality of evidence for the role of phosphorus for health-related outcomes as a basis for setting and updating dietary reference values (DRVs) for the Nordic Nutrition Recommendations (NNR) 2023 (Box 1).

Box 1 Background papers for Nordic Nutrition Recommendations 2023

- This paper is one of many scoping reviews commissioned as part of the Nordic Nutrition Recommendations 2023 (NNR2023) project (7)
- The papers are included in the extended NNR2023 report, but, for transparency, these scoping reviews are also published in Food & Nutrition Research
- The scoping reviews have been peer reviewed by independent experts in the research field according to the standard procedures of the journal
- The scoping reviews have also been subjected to public consultations (see report to be published by the NNR2023 project)
- The NNR2023 committee has served as the editorial board
- While these papers are a main fundament, the NNR2023 committee has the sole responsibility for setting dietary reference values in the NNR2023 project

Phosphorus plays an essential role in several biological processes (1, 2). Phosphorus-containing compounds are involved in, for example, adenosine triphosphate (ATP) synthesis, signal transduction, cell structure, cellular metabolism, regulation of subcellular processes, maintenance of acid-base homeostasis, and bone mineralization. In biological systems, phosphorus is present as phosphate.

The total amount of phosphorus in the human body is 800–1,200 g, 85% of which is in the skeleton, and the rest evenly distributed in all tissues. Phosphate is the most abundant anion in the human body and comprises about 1% of the total body mass. It is predominantly an intracellular anion, and in the skeleton, phosphate is mostly complexed with calcium in the form of hydroxyapatite. In soft tissue and cell membranes, phosphorus exists mainly as phosphate esters and, to a lesser extent, as phosphoproteins and free phosphate ions. In the extracellular fluid, about one-tenth of the phosphorus content is bound to proteins, one-third is complexed to sodium, calcium, or magnesium, and the remainder is present as inorganic phosphate (1, 3). Serum inorganic phosphate (S-Pi) concentrations vary with age, with the highest concentration in infants. The concentrations decline toward adulthood, and the normal range in adults is 0.8–1.5 mmol/L (1).

Phosphorus occurs widely in foodstuffs, but the highest contents are in protein-rich foods, including meat, fish, eggs, dairy, legumes, whole-grain cereals, nuts, and seeds (4). In plant-based foods, phosphorus occurs in the form of phytate. Inorganic phosphate compounds, that is, phosphate salts as well as organic starch-based phosphates, are also used as food additives in various foodstuffs (4). Food additive phosphates are generally used for various technological purposes; they are abundant especially in processed meat products, cola beverages, and processed cheeses. They are also used in, for example, fish industry in some deep-frozen fish products. In the European Union, the legislation requires that any food additives used are listed as E codes or their names on the package (5). It is notable that absorbability of phosphorus differs between the foodstuffs, being highest from food additives followed by animal-based products and being lowest in plant-based products (6).

## Methods

This scoping review follows the protocol developed within the NNR2023 project (7). The sources of evidence used in the review follow the eligibility criteria described previously (8). PubMed was used for literature search with the following strategy: (“phosphorus, dietary”[MeSH Terms] OR “phosphorus”[MeSH Terms] OR ((“phosphate”[TI]) AND (Diet OR Dietary OR FOOD OR Nutrition OR Nutritional))) AND (“2011”[PDAT]: “2021/08/01”[PDAT]) AND (review[Publication Type] OR systematicreview[filter] or meta-analysis[filter]) AND Humans[Filter]. Additional relevant sources such as publications of European Food Safety Authority (EFSA) were also used. The search date was 1^st^ of August 2021. The PubMed search resulted in 533 results. There were no relevant *de novo* or qualified systematic reviews (SR) for setting DRVs and FDBGs.

## Physiology

Dietary phosphate is absorbed by the epithelium of the duodenum and jejunum in the small intestine via both passive diffusion, which depends on the amount of phosphorus in the intestine, and an active sodium-dependent process by sodium-dependent phosphate transporter IIb. The active absorption is stimulated by low phosphorus intake that causes a decrease in S-Pi concentration and thus stimulates 1,25-dihydroxyvitamin D, calcitriol (1,25(OH)_2_D) (9). One type of sodium-dependent cotransporter III, PiT1, may also play a role in the phosphate absorption regulation but seems not be affected by dietary phosphate intake very largely. When phosphorus-containing food is ingested and phosphorus is absorbed, S-Pi concentration increases immediately (10).

Approximately 80–90% of phosphorus is reabsorbed in the proximal tubules of the kidney by the sodium-dependent phosphate transporters 2b, 2c, and PiT2 (9). Generally, net absorption from a mixed diet has been reported to vary between 55 and 70% in adults and between 65 and 90% in infants and children, and the absorption tends to decrease while aging (10). There are several factors that affect phosphorus absorption: the total amount of ingested phosphorus, type of phosphorus (organic/inorganic), food origin (animal/plant-based), and the ratio of phosphorus to other dietary compounds, especially calcium (10).

The fibroblast growth factor 23 (FGF23)-Klotho system regulates phosphorus metabolism together with parathyroid hormone (PTH). After ingestion of phosphorus, both PTH and FGF23, are released, the former from the parathyroid glands and the latter from osteocytes and osteoblasts in bone (2). PTH and FGF23 are the most important hormones that regulate renal phosphate handling by reducing the activity of sodium-dependent phosphate transporters 2a and 2c (9). FGF23 is a phosphatonin that requires the cofactor, Klotho, to act and to be able to bind to FGF23 receptor. Klotho, which is a single pass trans-membrane protein existing in different isoforms, is found in the kidney and in some other tissues such as the parathyroid glands. When dietary phosphorus intake increases, FGF23 and PTH are secreted causing a phosphaturic effect, whereas the concentrations of 1,25(OH)_2_D decrease, less phosphorus is absorbed from the intestine, and the S-Pi concentration is reduced (2). It has been suggested that PTH is mainly responsible for the increased phosphorus excretion after acute ingestion of phosphorus rather than FGF23 (9). Lately, a role of FGF receptor 1 (FGFR1) and the gene product of Galnt3 in increasing FGF23 in response to high phosphorus intake have been suggested in animal studies (11). Several other factors also affect phosphate reabsorption, including estrogen, insulin, growth hormone, thyroid hormone, and other phosphatonins, such as matrix extracellular phosphoglycoprotein and secreted frizzled protein-4 (9).

Calcium is also an important factor contributing to phosphorus metabolism. Serum calcium concentration affects the synthesis of 1,25(OH)_2_D, mediated by FGF23 and PTH. 1,25(OH)_2_D indirectly regulates the calcium-sensing receptor, which modulates secretion of PTH in parathyroid cell caused by calcium (2). There is a homeostatic balance in calcium and phosphate metabolism, in which 1,25(OH)_2_D and PTH are involved. For example, when S-Pi concentration increases, the excretion of phosphorus in the kidneys increases, and the absorption from the gut and release of phosphate from bone decrease. By the same mechanisms, the absorption of calcium from the gut and the release of calcium decrease, but the excretion of calcium in the kidney decreases. This leads to a decrease in S-Pi concentration, but the serum calcium concentration remains intact. Calcium and phosphorus are both required for bone mineral deposition and maintenance. For maintaining bone health, a dietary molar calcium to phosphorus ratio is suggested to be 1:1 (1.5 to 1 in mg), whereas a ratio below that may disturb mineral metabolism and contribute to bone loss (12).

The chemical composition of foodstuffs affects the absorbability of phosphorus (6, 13). Both human (*in vivo*) and *in vitro* studies have shown that absorbability of phosphorus differs between the foodstuffs. Absorbability of phosphorus is high from processed, phosphate additive-containing foods, such as processed meats, baking powder-containing bakery products, processed cheeses, and cola drinks, even 100%. Phosphorus absorbability from animal-based products is around 40–60%. In contrast, absorbability of phosphorus from plant-based products is low, 40% or less especially in legumes and seeds. In cereals, phosphorus becomes more bioavailable during processing when, for example, the bread is fermented, and phosphorus from phytate is released as absorbable, inorganic phosphorus through the action of microbial phytase (6). The human body does not secrete phytase, thus, for phosphorus to be available for absorption, phytate has to be degraded by phytase through food processing (fermenting) (14). Due to differences in absorbability, more plant-based diets and avoidance of phosphorus additives have been recommended for kidney patients, for whom controlling phosphate intake is crucial to maintain S-Pi concentrations (15).

## Assessment of nutrient status

Usually, phosphate is measured in blood and urine. S-Pi concentration increases for a short time after ingestion of a meal containing phosphorus and due to the earlier described homeostatic mechanisms. Afterwards, the concentration decreases and remains within a relative narrow range (0.8–1.5 mmol/L) (1). Because of renal regulation, there is not much modification in the concentrations even during a high variation of phosphorus intake levels (10). It has been suggested that FGF23 or PTH could be used as surrogate markers of high phosphorus intake, and their increased concentrations could be the main reasons for the potential health effects of high P intake (9, 16, 17). PTH and FGF23 are both known to increase with high dietary P intake (9). However, PTH is affected also by vitamin D status (serum 25-hydroxyvitamin D concentration), serum ionized calcium concentration, as well as S-Pi, which impairs the use as a status biomarker (10). Also, FGF23 concentrations are affected by other factors such as calcium and iron intake and metabolic health disturbances (17). There is an innate circadian variation in S-Pi, FGF23, and PTH (12). A continuous measurement of S-Pi over the day has been suggested to better describe long-time dietary phosphorus load (16) because fasting S-Pi reflects the homeostatic level better than postprandial measurements (18), and inter-individual variation is low (17).

Among healthy individuals, phosphorus is excreted through the kidney, and urinary phosphate excretion may reflect dietary intake. Nevertheless, various hormonal factors involved in calcium and phosphorus metabolism affect urinary phosphate excretion, which impairs the use as intake and status biomarkers (10). Finding no associations in observational studies between dietary phosphorus intake and urinary phosphate excretion or S-Pi concentrations can also be due to differing absorbability of different phosphorus sources as well as incomplete food composition databases and the use of various dietary assessment method (16, 19).

The EFSA Panel on Dietetic Products, Nutrition and Allergies (NDA) not only concluded in 2015 (10) that there is no reliable biomarker of phosphorus intake and status that could be used for setting the requirement for phosphorus, but also mentioned that the role of FGF23 and other phosphatonins should be further studied as potential biomarkers for phosphorus status (10).

## Dietary intake in Nordic and Baltic countries

The mean dietary phosphorus intake in the Nordic countries exceeds the 2012 Nordic nutrition recommendations [recommended intake (RI) in NNR2012: 470–600 mg/day (20)] 2- to 3-fold in all age groups (21). Mean intakes are above 1,000 mg/day in all age groups, ranging up to 1,900 mg/day. In the Baltic countries, phosphorus intakes are slightly lower; however, adequate and the mean intakes are higher among men than women. Phosphorus intakes per MJ in the Nordic countries are approximately 160–190 mg/MJ/day in Finland, Sweden, and Iceland (no data from Norway and Denmark). In the Baltic countries, intakes per MJ are 120–160 mg/MJ/day. Factors related to dietary surveying, food composition databases, and calculation procedures differ between the countries and should be taken into account when comparing the results (21). Food composition databases do not contain updated and complete information on food additive phosphates (12).

The main sources of phosphorus in the Nordic countries are dairy products, cereal products, and meat products (22–25). Sources of protein are often rich in phosphorus, and dairy products are good sources of calcium. Food additives can be a significant source of phosphorus, but their contribution to the total phosphorus intake in the Nordic countries is unknown. Generally, it has been estimated that food additives can contribute significantly to the total phosphorus intake in the Western countries (12). In the European Union, the legislation requires that any food additives used are listed as E codes or their names on the package (5). A recent Finnish study on food package labelings showed that one-third of the reviewed processed foodstuffs contained food additive phosphates; however, the amounts are unknown (26).

## Health outcomes relevant for Nordic and Baltic countries

### Phosphorus deficiency

Hypophosphatemia leads to skeletal defects (impaired bone mineralization, rickets, and osteomalacia) and problems with the nervous system, muscle tissue, and kidney function (27). Refeeding syndrome is one cause of hypophosphatemia (28). Diet-related phosphorus deficiency is very rare because the share of phosphorus in the foodstuffs is abundant, and absorption is very efficient. Phosphorus deficiency is generally related to metabolic disorders. Although vitamin D deficiency or resistance decreases phosphorus absorption, hypophosphatemia due to low intestinal absorption is rare and only becomes apparent when phosphorus deprivation has continued for a long time, such as in the case of diarrhea (27).

### Consequences of high phosphorus intake and increased serum phosphate concentrations

#### Toxicity

EFSA considered phosphates to be of low acute oral toxicity, and there is no concern with respect to genotoxicity and carcinogenicity (29). No effects were reported in developmental toxicity studies. However, high use of phosphate supplements can cause gastrointestinal symptoms (osmotic diarrhea, nausea, and vomiting).

#### Effects on bone and vascular system

Renal patients have impaired mineral metabolism, and they are unable to excrete phosphorus properly, causing hyperphosphatemia and resulting in, for example, vascular calcification if not treated with phosphorus-restricted diets (9). Excessive phosphorus intake and increased S-Pi concentrations can also be harmful for the bone and vascular systems in healthy subjects. High S-Pi concentration is one factor that triggers the differentiation of vascular smooth muscle cells into osteoblast-like cells (2). Even transient increases of S-Pi concentrations, for example, postprandial hyperphosphatemia, can promote endothelial dysfunction (30).

High S-Pi concentration has been associated with vascular calcification as well as cardiovascular and all-cause mortality in the general population in meta-analyses (31, 32). S-Pi concentrations increase quickly after ingestion of phosphorus-containing food, with an increase in serum PTH concentrations. Thus, health outcomes related to S-Pi are also important even though fasting S-Pi may not be the best way to measure the dietary phosphorus load (see Assessment of nutrient status section). Adverse outcomes related to S-Pi are more well-established among chronic kidney disease patients: two meta-analyses have shown that high S-Pi concentrations associate with progression of the disease, vascular calcification, and osteopenia as well as with mortality (33, 34). However, there is a lack of concluding data on the effects (meta-analysis) regarding phosphorus intake and many health outcomes. The only systematic review regarding phosphorus intake and excretion did not find a consistent association between total dietary phosphorus intake and blood pressure (35). No meta-analysis related to dietary phosphorus (or food additive phosphate) and other adverse outcomes related to vascular health is available. Some cross-sectional studies have shown associations with left ventricular mass, carotid intima-media thickness, and mortality (36–38).

Adverse effects of high dietary phosphorus on bone health are mainly based on the elevated PTH concentration, thus mild and transient secondary hyperparathyroidism (18), but the evidence is inconclusive. High-phosphorus diets can cause abnormalities in bone and mineral metabolism, especially when dietary calcium-to-phosphorus ratio is low, thus possibly leading to low bone mineral density (18). Some cross-sectional studies have indicated that phosphorus intake, especially as food-additive phosphorus, is associated with higher PTH concentrations and could be deleterious to bone health (27).

## Requirement and recommended intakes

### Main results

Phosphorus deficiency related to low intake is very rare. The main phosphorus-related health outcomes concern high phosphorus intake with adverse effects on kidney, bone, and cardiovascular health; however, more evidence is needed.

### Recommendations and reasoning

The RI for phosphorus in the previous NNR from 2012 (20) was 600 mg/day, which is based on the molar calcium to phosphorus ratio 1:1. For adolescents, the recommendation was slightly higher—700 mg/day because of a higher requirement of phosphorus due to bone accretion.

In 2015, the EFSA NDA Panel provided a scientific opinion on setting DRVs for phosphorus (10). The Panel considered data from balance studies on losses of phosphorus from the body and intestinal absorption for possible use in a factorial approach, and studies on phosphorus intake and long-term health outcomes (bone health, cancer-related outcomes, and evidence related to all-cause mortality and cardiovascular outcomes). It was concluded that the data were insufficient to be used to derive DRVs for phosphorus. Based on different scenarios, they chose the molar whole body calcium to phosphorus ratio ranging from 1.4:1 to 1.9:1 to set DRVs based on the calculation of amount of phosphorus, and the lower bound was used to set Adequate Intake (AI). They concluded that AI of phosphorus should be 550 mg/day for adults and both sexes (10). This was also applied to pregnant and lactating women because the calculation takes into account the adaptive changes in phosphorus metabolism during those periods. For adolescents, an AI of 640 mg/day was set, while 160 mg/day was set for infants (7–11 months) and between 250 and 640 mg/day for children. However, the Panel considered that the current data were not sufficient to derive average requirements and population reference intakes. After that some more data on blood pressure have been published (35), but they do not provide new evidence to be considered for setting an RI for phosphorus in NNR2023.

In the latest update on dietary reference intakes in the US that was published in 1997, the estimated average requirement (EAR) for phosphorus was set at 580 mg/day for both men and women aged 19–70 years based on the lower end of the adult S-Pi range (0.87 mmol/L), which was considered the best available EAR for adults (39). The recommended dietary allowance (RDA) was set at 700 mg/day for both sexes. For adolescents 9–18 years of age, the RDA was set at 1,250 mg phosphorus per day based on both dietary data and estimated additional needs during growth (39). In 2016, new evidence since 2012 to prioritize updates for dietary reference intakes was reviewed, and the nutrient review group concluded that there was insufficient new evidence to assign a high priority to a comprehensive systematic review on phosphorus (40).

There are no substantial new data since then to indicate that the RI values in NNR2012 (20) should be changed. This intake level adheres to the view that an equimolar relationship between calcium and phosphorus is used as a basic principle for recommendations (1 mmol calcium = 40 mg and 1 mmol phosphorus = 30.9 mg). The RI values for children should also be maintained to be based on the same considerations.

In 2005, EFSA NDA Panel concluded that there was no sufficient data available to establish the tolerable upper intake levels (UL) for phosphorus intake (41), but the Panel stated that normal healthy individuals can tolerate phosphorus intakes up to at least 3,000 mg/day without adverse systemic effects. This EFSA value was applied in the NNR2012 (20). In 2015, EFSA did not give suggestions for UL for children, lactating, or pregnant women (10). On the contrary, in the US (39), an UL has been set for total intake of phosphorus for children aged 1–8 years at 3,000 mg/day and 4,000 mg/day for children aged from 9 years and adolescents as well as adults (with an exception of 3,000 mg/day for older adults), respectively. The IOM’s UL for pregnant women is 3,500 mg/day and for lactating women, it is 4,000 mg/day (39).

The fact that the UL for phosphorus may be considered is new available data on high intake. Based on EFSAs recent reevaluation, the safety of particular food additive phosphates (E 338–341, E 343, and E 450–452), a group acceptable daily intake (ADI) for phosphates expressed as phosphorus of 40 mg/kg body weight (bw) per day, was set (for a 70-kg individual, it is 2,800 mg/day) (29). The ADI was exceeded in the estimated exposure scenarios for infants, toddlers, and other children at the mean level, and infants, toddlers, children, and adolescents at the 95th percentile, but there was no safety concern in infants below 16 weeks of age consuming formula and food for medical purposes. In the scenarios, phosphate exposure from food supplements exceeded the proposed ADI (29). However, there is no information whether phosphorus supplements are used in remarkable amounts in the Nordic countries. Data from Finland show that 36% of the foodstuffs on the market contained food additive phosphates, and most of them were inorganic phosphates (26). However, no quantitative data of the amounts are available, and the manufacturers are reluctant to provide the data. Based on the new data, an UL based on ADI should be applied, and especially, an UL for vulnerable groups with lower bodyweight (infants, toddlers, children, and adolescents) should be considered.

## Limitations of the scoping review process

There are limitations in the scoping review process because studies on dietary phosphorus intake and specific health outcomes are rare, and there is a lack of SRs and meta-analyses of the topic. There were no qualified or de novo SRs available for phosphorus intake that could be used to set DRVs. Evidence is mainly based on observational studies and short-term trials. High-quality RCTs are needed.

## Data gaps for the future research

Effects of phosphorus on health may depend on the source from which it is ingested. However, there is a growing need to develop research methods by which phosphorus bioavailability can be taken into account. Data on the use of food additive phosphates by the food industry and updated chemically analyzed information on phosphorus contents in foodstuffs are also needed. When assessing the phosphorus contents in foodstuffs, natural phosphorus and food additive-derived phosphorus cannot be distinguished analytically (29), and thus, phosphorus contents in food composition databases are reported as total phosphorus. Moreover, in food composition databases, phosphorus contents of the recipe-based foodstuffs are calculated based on nutrient contents of raw materials, and not on chemically analyzed values (e.g. 19). Thus, the amounts of food additive phosphates usually have not been considered. Because phosphorus intake cannot be measured correctly, it also impairs the associations between phosphorus intake and potential health outcomes. New dietary assessment methods, such as untargeted metabolomics, can provide some answers to these questions in the future. It is notable that in 2015, EFSA concluded that there was no sufficient data from balance studies on losses of phosphorus from the body and intestinal absorption for possible use in a factorial approach, and studies on phosphorus intake and long-term health outcomes to be used to derive DRVs for phosphorus (10). They used other methods related to whole-body calcium to phosphorus ratio but considered them to be insufficient to derive average requirements and population reference intakes. Thus, good-quality research data on, especially, long-term health outcomes and risk of chronic diseases are needed.
